# Sensitivity of core-level spectroscopy to electrostatic environments of nitrile groups: An *ab initio* study

**DOI:** 10.1063/1.5003404

**Published:** 2017-09-25

**Authors:** Abid Hussain, Nils Huse, Oriol Vendrell

**Affiliations:** 1 Max Planck Institute for the Structure and Dynamics of Matter, Center for Free Electron Laser Science, 22761 Hamburg, Germany; 2 Department of Physics, University of Hamburg and the Hamburg Centre for Ultrafast Imaging, 22761 Hamburg, Germany; 3 Center for Free-Electron Laser Science, DESY and The Hamburg Centre for Ultrafast Imaging, 22761 Hamburg, Germany; 4 Department of Physics and Astronomy, Aarhus University, Ny Munkegade 120, DK-8000 Aarhus C, Denmark

## Abstract

*Ab initio* quantum chemistry calculations have been performed to probe the influence of hydrogen bonding on the electronic structure of hydrogen cyanide (HCN). Our calculations determine the origin of nitrogen-specific Raman spectral features from resonant inelastic X-ray scattering occurring in the presence of a water molecule and an electric dipole field. The similarity of the two interactions in altering the electronic structure of the nitrogen atom differs only in the covalent contributions from the water molecule. The CN stretching mode as a structural probe was also investigated to study the electronic origin of the anomalous frequency shift of the nitrile group when subjected to hydrogen bonding and an electrostatic dipole field. The major changes in the electronic structure of HCN are electrostatic in nature and originate from dipole-dipole interactions. The relative shifts of the CN stretching frequency are in good agreement with those experimentally observed.

## I. INTRODUCTION

Noncovalent interactions are important for the functionality of biomolecules and play a major role in the structure and dynamics of a wide class of molecular systems.^1–13^ Understanding the influence of noncovalent interactions, namely, electrostatic, hydrogen bond (HB), and van der Waals interactions, on the electronic structure poses a considerable challenge and has been the topic of extensive studies in the protein structure and dynamics.^14,15^ A large number of studies on hydrogen bonding at the molecular level have shed light on spectroscopic changes taking place in these complex systems.^16–21^ Among the noncovalent interactions, hydrogen bonding is very directional, of short range, stronger than van der Walls interactions, and crucial for proton transfer.

Of the many accepting groups in hydrogen bonds, we have a particular interest in the nitrile group for reasons that we detail below. There are several studies on donor-acceptor interactions of nitrile groups with different solvents to determine electrophilicity and solvation properties. The nitrile group is a weak HB acceptor which is sensitive to hydrogen bonding, solvent polarity, and surrounding electric fields.^9,15,22–32^ The lone electron pair on the nitrogen atom of the nitrile group provides the site for hydrogen bond acceptance of a proton donor such as water, leading to an anomalous shift of the CN stretching vibration to higher energy. This bond stiffening is unusual because almost all other stretching vibrations of HB acceptor groups (with the exception of diazo and azide groups for instance) display bond softening upon hydrogen bonding.^33^ Studies of protein processes have made use of this shift by incorporation of nitrile-derivatized amino acids.^14,15,34,35^ Such non-natural amino acids cause only small perturbations in the native structure of proteins and constitute spectrally isolated and well distinguishable vibrational probes for electrostatic environments, charge transfer and migration, and structural dynamics of local protein environments. Accordingly, a number of reports have been published on the use of nitriles as a probe of protein folding and unfolding dynamics, electric field effects on active sites in peptides and proteins, mechanisms of biological information transfer by nucleic acids, and other biological processes.^36–39^ It is therefore important to understand and gauge the electronic and structural response of nitriles to electrostatic and hydrogen bond interactions on the atomic level.

The hydrogen cyanide (HCN) molecule as the simplest molecule containing a nitrile group represents a good starting point for establishing basic principles of interaction of the nitrile group with its environment because the localized nature of excitations of the nitrile group allows drawing direct conclusions from HCN to other similar nitrile systems. Several prior studies on HCN^40–45^ exist, but a combined systematic study of molecular and element-specific electronic structure using vibrational and X-ray methods, respectively, is missing. Such an approach would generally highlight the interplay of electronic and structural degrees of freedom. Over the last decade, developments in the fields of synchrotron radiation research and sample delivery opened up new scientific avenues for more advanced core-electron spectroscopy, allowing for investigation of complex chemical reactions and local effects in heterogeneous environments due to surrounding solvent molecules, interfaces, or electrostatic fields from charge distributions generated by, for instance, protein side chains. *Ab initio* methods help establish a connection between physical observables and electronic structure to guide the interpretation of spectral features observed experimentally. Recent X-ray spectroscopic studies of acetonitrile, the next simplest nitrile-containing molecule, revealed orbital anisotropy and nuclear relaxation upon core excitation.^46,47^ *Ab initio* methods have been employed to analyze solvent-solute interactions energetically, decomposing them into their contributing parts.^17,18,20,21,48^

The nitrogen K-edge provides an element-specific resonance with an initial state having a highly localized 1 s-electron that allows studying the nitrile group in the presence of protic solvents like water and surrounding electrostatic environments directly at the interaction site. As structural and electronic changes generally manifest sensibly in the infrared and the ultraviolet spectral range, respectively, it is of general interest to understand and link complementary local structural and electronic probes. Such insight could shed light on the origins of the anomalous frequency shift of the stretching vibration of the nitrile group (also found in the corresponding vibrations of the diazo, and azide groups), which has not been satisfactorily explained on the level of atomic charge distributions. This work investigates the response of HCN to model electrostatic environments and relates this response to electronic and structural changes detectable in nitrogen-1 s resonance Raman spectra in the ultraviolet (more commonly known as resonant inelastic X-ray scattering or RIXS) and frequency shifts of the CN stretching vibration in the infrared. We compare computed nitrogen-1 s RIXS spectra of HCN to the response of the HCN-water complex and analogous calculations for dipoles of varying strength in axial and equatorial configuration in order to separate and classify the observed spectral features arising from covalent and purely electrostatic interactions of HCN and water.

**FIG. 1.**
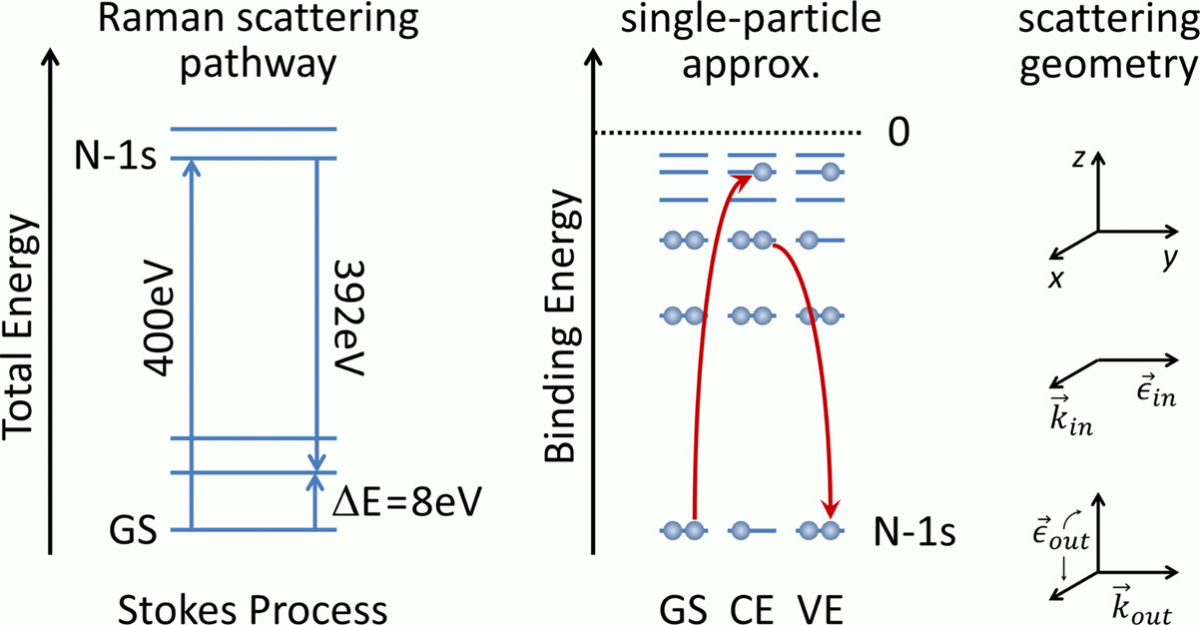
Schematic representation of Raman processes exploiting a nitrogen-1 s core-level excitation. The Stokes process will promote the system into an excited state. Within the single particle picture, the RIXS process that starts from the electronic grounds state (GS) can be subdivided into a core-level excitation (CE), lasting a few femtoseconds, and a subsequent relaxation into a valence-excited (VE) state. A similar excited state could be reached with direct (dipole) excitation but with different quantum mechanical selection rules.

RIXS is an element-specific form of resonance Raman scattering, in which a core-excitation of a particular element is exploited to achieve chemical specificity while obtaining Raman spectra of molecular excitations. The Raman process is not limited to vibrational excitations but can be employed to measure element-specific electronic spectra in the visible and ultraviolet as Fig. 1 illustrates. An X-ray-initiated Stokes process can result in an electronic excitation in the ultraviolet, while an anti-stokes process in the multi-eV range cannot be effected thermally under standard temperature and pressure conditions. However, anti-Stokes scattering pathways have been exploited using short light pulses (promoting the molecular system into an electronically excited state).^49^ As customary in Raman spectroscopy, the energy difference between incoming and inelastically scattered photon (the energy loss) is plotted which provides the studied system's vibrational and valence electronic excitations.

### A. Computational details

The equilibrium geometries of HCN and the HCN-water complex were optimized at the second order Møller-Plesset theory (MP2) level with the Dunning correlation consistent basis set aug-cc-pvtz.^50^ The results of the vibrational frequency analysis reveal that the computed structures are those of true energy minima. Geometries were optimized using the Gaussian 09 suite^51^ of programs.

**FIG. 2.**
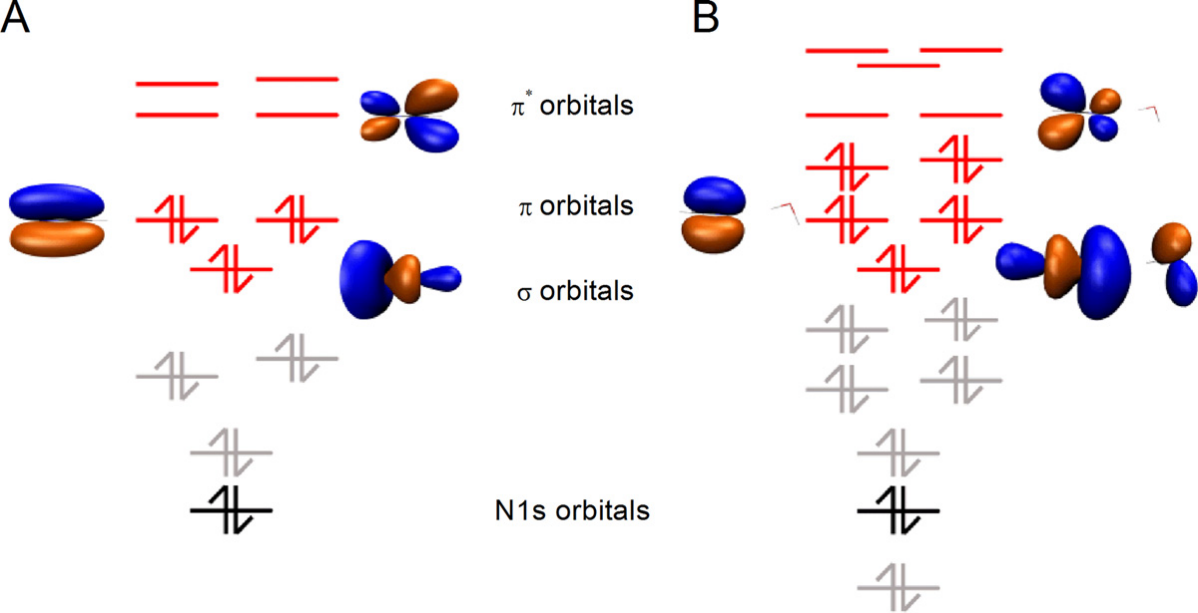
Energy level diagram of HCN (a) and the HCN-water complex (b). Molecular orbitals (MOs) marked in red and black are included in the active spaces for RIXS computations. These orbitals are partitioned into the two subspaces RAS2 (red occupied and virtual orbitals) and RAS3 (black nitrogen-1 s core-level). For orbitals involved in dominant transitions, isodensity surfaces are plotted.

RIXS spectra were calculated with the first-principles multi-configuration restricted active space self-consistent field (RASSCF) approach^52^ using the Dunning correlation consistent basis^50^ set aug-cc-pvtz for all atoms. No symmetry is imposed for the RIXS calculations. The active space of HCN comprises 8 electrons distributed in 8 orbitals having characters of *π*, *σ*, and π*. For the HCN-water complex, the active space comprises 12 electrons distributed in 11 orbitals with 8 of them having the same character as those of the isolated HCN molecule. These two orbital sets are shown in Fig. 2.

For the calculation of valence states, state averaging with 13 states is performed, whereas for the calculation of core-hole states the energies of three states are averaged in the self-consistent calculation. The choice of 13 states includes energy losses up to 14 eV beyond which only very weak transitions were found in HCN for a higher number of valence states. Furthermore, no experimentally discernable features exist in acetonitrile, which features a very similar RIXS spectrum.^46,47^

All orbitals without occupational constraints are kept in the active subspace RAS2, while core electrons are placed in the active subspace RAS3, where at most one electron is excited (thus suppressing configurations with a doubly filled or excited nitrogen core orbital). Grouping the excitations in such a way avoids the presence of irrelevant low-energy configurations during the calculation of core excitations and prevents the variational collapse of the wave function during the RASSCF calculations. Dynamic correlations are included by the multistate second-order perturbation theory (MS-RASPT2)^53–55^ using 0.25 Hartree for the ionization potential electron affinity (IPEA) shift and an imaginary shift of 0.3 Hartree to avoid intruder states. The dipole transition moment between any two RASSCF wave functions is obtained by restricted active space state-interaction (RASSI)^56,57^ calculations. RASSCF/RASSI calculations were performed using the MOLCAS 7.8 suite.^58^

RIXS intensities are computed within the static approximation (without propagation of the system while in the core-excited state) by the Kramers-Heisenberg relation using matrix elements obtained by the state interaction framework over RASSCF wave functions.^52^ We have chosen a scattering geometry that is customary for solution-phase RIXS experiments, with photons incident on and scattered from the sample having k-vectors and polarizations $\left({\vec{k}}_{in},{\vec{\epsilon }}_{in}\right)$ and $\left({\vec{k}}_{out},{\vec{\epsilon }}_{out}\right)$, respectively, as shown in the right of Fig. 1. A liquid jet would have a velocity ${\vec{v}}_{jet}$ in the negative z-direction (flowing downward in Fig. 1). This perpendicular arrangement of incoming and outgoing photon k-vectors suppresses the elastic peak to acceptable levels allowing for the largest signals to arise from inelastically scattered photons. Additional polarization-sensitive detection can easily be implemented if required.^59^ The scattering cross-section (in atomic units), averaged over all orientations of the molecule and integrated over all directions and polarizations, is^59,60^

$$\sigma^{i\to f}=\frac{8\pi \omega_{out}^{3}\omega_{in}}{9c^{4}}\sum_{\rho \lambda }{\left|{\left(\alpha_{fi}\right)}_{\rho \lambda }\right|}^{2}$$ (1)

where ${\left(\alpha_{fi}\right)}_{\rho \lambda }$ is the polarizability tensor element describing inelastic scattering according to

$${\left(\alpha_{fi}\right)}_{\rho \lambda }=\sum_{n}\frac{〈f\left|T_{\rho }\right|n〉〈n\left|T_{\lambda }\right|i〉}{E_{n}-E_{g}-\hbar \omega_{in}-i\Gamma_{n}}.$$ (2)

The integral RIXS cross-section coincides with the scattering cross-section at the so-called “magic” angle (54.74^°^) between the polarization of incident and scattered photons.^59^ In the above equations, *ω_in_* and *ω_out_* are the angular frequencies of the incident and outgoing scattered photons. *E*_*i*,*n*,*f*_ corresponds to the energies of the initial, intermediate, and final states, respectively. The subscripts *ρ* and *λ* denote x-, y-, and z-components of the electric dipole transition operator T. The lifetime broadening of the intermediate states is denoted by Γ and c is the speed of light (∼137 in atomic units). Equation (2) describes a resonant process, while non-resonant terms are irrelevant in this case and have been omitted. Interference effects of intermediate states decaying to the same final state are characteristic for this type of spectroscopy.^59,61^

Orbital energies were used to rationalize the variation in electronic eigenstate energies as a function of dipole strength. Orbital energies correspond to Hartree-Fock ground electronic state calculations, and for the multiconfigurational electronic wavefunctions, it was in all cases possible to identify a leading configuration and a well defined excitation character in terms of those single-particle functions. In order to correlate structural and electronic degrees of freedom, the potential energy surface (PES) of CN bond stretching in HCN in the presence of dipoles of varying strengths is computed by changing the CN bond length within the small (i.e., harmonic) variation limit. The CN bond length is varied in such a way that the center of the CN bond and the length of the CH bond are fixed. From the computed curvature of the PES for each dipole configuration and strength, the frequency of the CN stretching vibration is calculated according to $\nu_{CN}={\left(2\pi \right)}^{-1}\sqrt{k/\mu }$, with *k* as the molecular force constant derived from the PES curvature by finite differences and *μ* as the reduced mass of the C and N atoms. For the strongest dipole, we constructed a two-dimensional section of the PES along the CH and CN stretching coordinates and diagonalized the corresponding Hessian matrix to obtain the vibrational frequencies and normal modes. Owing to the small coupling between the two stretching vibrations, treating the CN stretching mode as an isolated vibration does not alter the results.

## II. RESULTS AND DISCUSSIONS

### A. Isolated HCN

**FIG. 3.**
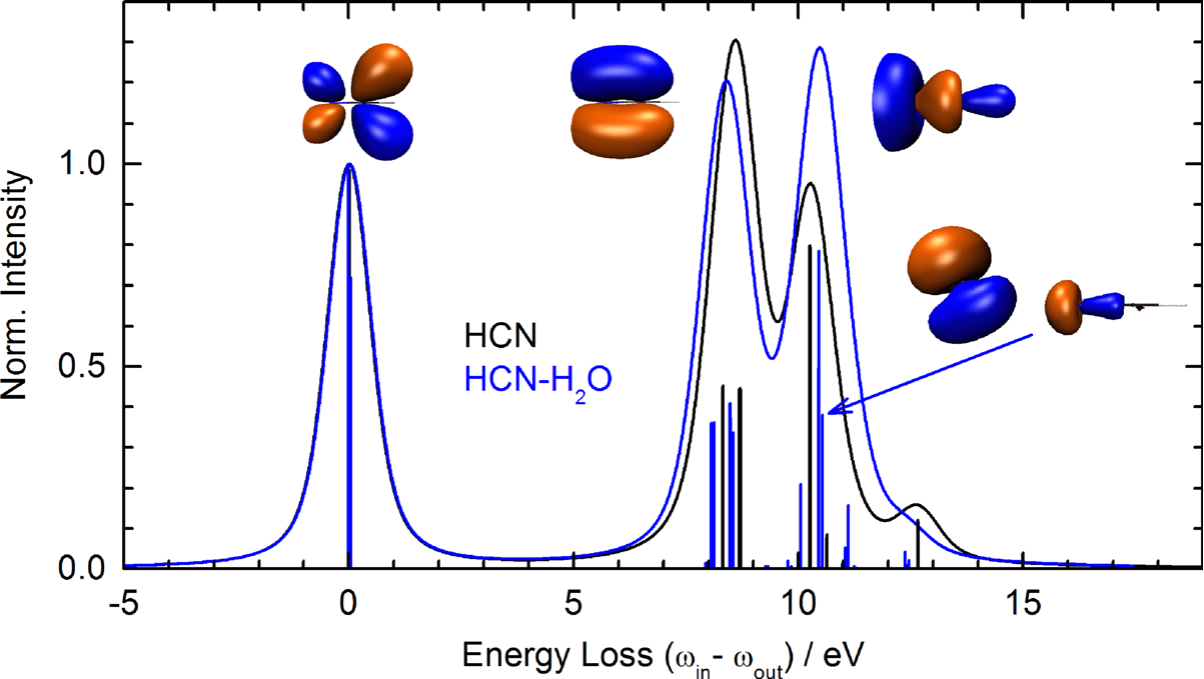
Loss spectra of HCN (black) and hydrogen bonded HCN (blue). Lineshapes are the same for both calculations. The isosurfaces represent the orbitals from which the core-hole is predominantly filled, the LUMO π^*^, the HOMO *π*, and the lone-pair *σ* orbital. The HCN-water complex features additional transitions between 10 and 11 eV from hybridized HCN-water orbitals as indicated by the blue arrow.

Spectroscopic studies on isolated HCN and its clusters have reported the electronic and molecular structure.^40–42,62^ This work focuses on exploring the sensitivity of nitrogen-1 s RIXS as a probe of weak to intermediate electrostatic and HB interactions by identifying the spectral features of valence charge distributions with atomic detail and correlating them with structural probes. The calculated RIXS spectra of HCN at the nitrogen K-edge are obtained at 400 eV incident photon energy where the maximum absorption is predicted. The corresponding loss spectra are presented in Fig. 3 (black line) along with the lowest unoccupied molecular orbitals (LUMOs) involved in the dominant core-level transitions. Our theoretical assignment attributes the elastic peak at zero loss energy to the relaxation of the core-level excitation from a π^*^ LUMO subsequent to N-1 s excitation. The first inelastic peak is observed at a loss energy of 8.5 eV. It is due to a relaxation originating from two occupied MOs of *π* character. The next higher RIXS feature manifests at a loss energy of 10.22 eV. It stems from valence-to-core transitions originating from molecular orbitals of mainly *σ* character. The spectral feature observed at about 12.6 eV corresponds to a Rydberg state of very mixed character with major contributions from *π* orbitals. A similar theoretical feature in acetonitrile is characteristic of an isolated molecule which vanishes in solution.^21,47^

### B. HCN-water complex

Next, we investigate the N-1 s RIXS spectra of HCN interacting with one water molecule. The loss spectrum of hydrogen-bonded HCN (blue line) is also plotted in Fig. 3. Clearly, all the major spectral features for isolated HCN are preserved in the relatively weakly interacting complex. The elastic peak originates from the two π^*^ orbitals populated upon core-level excitation and core-hole relaxation without photon energy loss. The relaxation from initially occupied *π* orbitals is now shifted to slightly lower energy loss of 8.3 eV. The largest change occurs at the peak originating from the relaxation of the lone pair electrons in the *σ* orbital, resulting in a loss energy transition of 10.44 eV. Owing to hydrogen bond formation, the resonant transition occurs at a lower emission energy, i.e., larger loss energy—a clear indication of orbital stabilization. In the same range of the loss spectrum additional transitions appears upon hydrogen bonding (cf. blue arrow in Fig. 3), which enhances the peak at 10.5 eV. These transitions originate from orbitals of the mixed HCN-water character, pointing to orbital covalency between the two molecules. We conclude that even weak hydrogen bonds with lengths as large as *d_NO_* = 3.05 Å are not entirely electrostatic in nature.

The increased binding energy of the lone-pair electrons can be correlated with a stiffened CN bond as frequency calculations on the electronic ground state PES show: The CN stretching frequency is predicted to shift from 2200 cm^–^^1^ for isolated HCN to 2206.5 cm^–^^1^ for the HCN-water complex, in agreement with results reported by Purcell, and is also found theoretically as well as experimentally in other systems containing nitrile groups.^63,64^

### C. HCN-dipole interaction

**FIG. 4.**
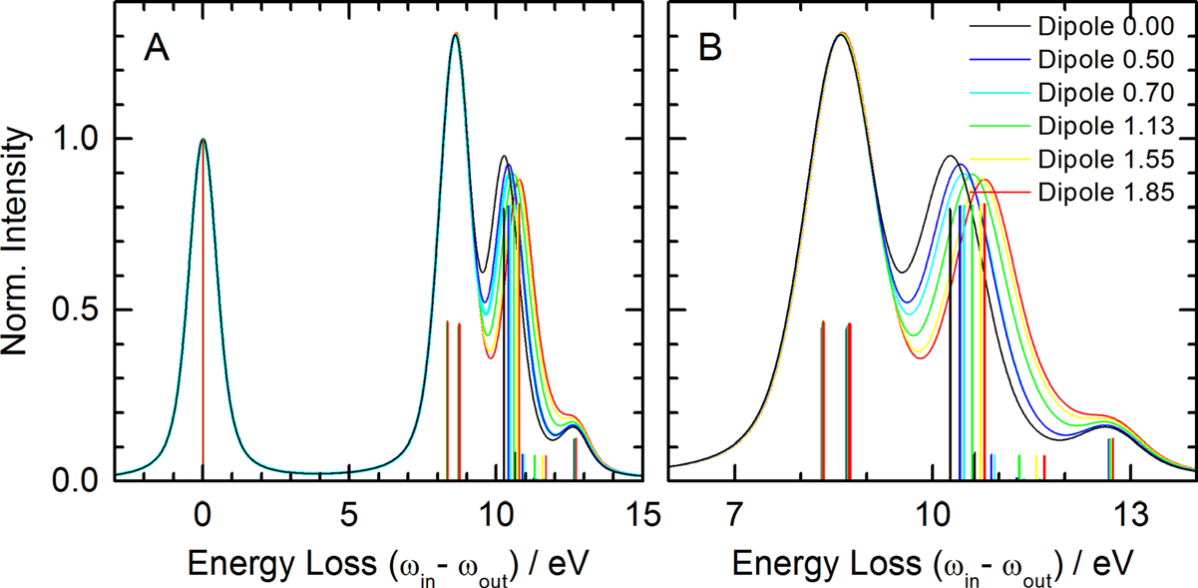
Simulated N-1 s loss spectra of HCN in the presence of an axial dipole of increasing strength as indicated in the legend. (a) Computed energy loss range. (b) Energy loss region with largest spectral changes. Vertical lines indicate transitions without broadening.

One aim of this study is to explore the response of the nitrile group to charge distributions. Moreover, the separation of electrostatic from covalent interactions in the observed spectral shifts can be attempted by placing a dipole of varying strength along the symmetry axis of an isolated HCN molecule. The positive charge of the dipole is thereby facing the nitrogen lone-pair at a distance of 2 Å, which is equal to the distance R(N ⋯ H) in the HCN ⋯ H_2_O complex (the corresponding hydrogen bond length is the donor acceptor distance of 3 Å). The effects of such a dipole on the loss spectrum of HCN are presented in Fig. 4. All spectral features have the same assignments as those of isolated and hydrogen-bonded HCN. It is clear from these spectra that the peaks resulting from the elastic scattering transition and those of the occupied *π* orbitals to the core-level do not show significant changes as a function of dipole strength. The major change is observed in the transitions involving the occupied *σ* orbital. These transitions exhibit a pronounced shift to higher energy with the increasing dipole strength, correlating with the stabilization of the *σ* orbital as already discussed for the HCN-water complex. This is a clear indication of the largely (but not entirely) electrostatic nature of weak hydrogen bonds formed with weak hydrogen bond acceptor groups such as the nitrile group interacting with a water molecule. Importantly, this result indicates that such a shift is universal rather than linked to the hydrogen bond donor that we chose to investigate. We conclude that such shifts can be used as quantitative probes of an electrostatic environment in more complex systems.

**FIG. 5.**
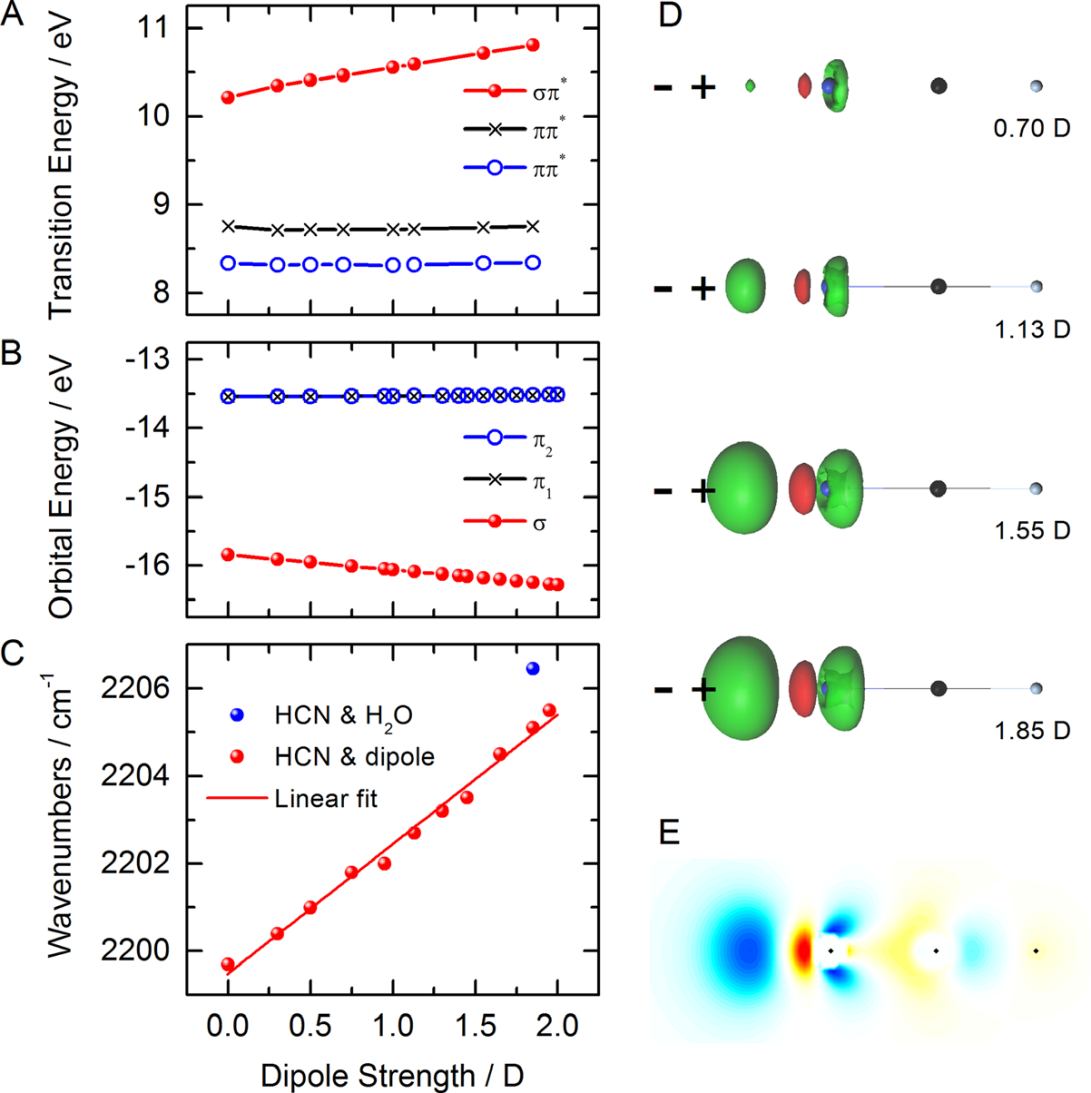
**(**a**)** Energies of valence excitation as a function of dipole strength. (b) Hartree-Fock orbital energies of the HOMO-*π* and HOMO-*σ* orbitals as a function of applied dipole strength. (c) Computed CN stretching frequency of HCN in harmonic approximation as a function of dipole strength. (d) Isodensity surfaces (at 0.005 value) of differential charge density between isolated HCN and HCN in the presence of an axial dipole. Green regions denote positive (increased) and red regions denote negative (decreased) charge density. (e) Cross-section of the differential charge density for the 1.85 D dipole. Blue and yellow to red areas represent increased and decreased charge density, respectively.

Further insight into the altered charge distribution of the nitrile group subjected to an axial dipole can be gained from the analysis of the dominant transitions and the Hartree-Fock-based orbital energies of the relevant highest occupied molecular orbitals (HOMOs). Both quantities are plotted in Figs. 5(a) and 5(b), respectively. The orbital energies of the *π* HOMOs show only small changes, while the energy of the *σ* HOMO orbital exhibits a linear dependence on the dipole strength. This increasing stabilization with a stronger dipole results in the lower emission energy of the transition from the core-excited (CE) to a valence-excited (VE) state (cf. Fig. 1) and therefore larger energy loss (i.e., increased binding energy) in the computed spectra of Fig. 4.

It is informative to examine the dependence of the CN stretching frequency on the dipole strength. The results, computed by variation of the CN bond distance, are plotted in Fig. 5(c). The CN stretching vibration shifts to higher energy with increasing dipole strength, signaling a stiffened CN bond. This behavior and the increasing *σ* orbital stabilization (directly manifesting in the loss spectra) can be related to the charge density difference between the isolated and the field-subjected HCN molecule. Differential isosurfaces for four dipole strengths are presented in Fig. 5(d). The charge density difference shows the expected shift of charge from the nitrogen atom towards the applied dipole. But the response of the nitrile charge distribution to a dipole or a hydrogen bond donor group is remarkable because it not only results in a shift of the lone-pair electrons of the nitrogen atom toward the dipole/HB donor (thereby inducing a dipole), but it actually creates an additional charge density modulation between the nitrogen and the carbon atom that polarizes the HCN molecule. This density modulation is shown in Fig. 5(e). Its shape resembles the *σ*-orbital of the CN triple bond (Fig. 3). It appears that the induced lone-pair dipole polarizes the *σ*-orbital of the CN triple bond along its nodal lines which can also affect bonding beyond the carbon atom. This effect, we conclude, holds responsible for the computationally observed bond shortening by a fraction of a picometer which, according to the slope of the linear fit in Fig. 5(c), shifts the CN stretching frequency higher by about 3 cm^–1^/*D*. We also find the same charge density modulation in the HCN-water complex. This finding provides an atomically resolved explanation of the well-known anomalous blue-shift of the nitrile stretching frequency as observed, for instance, in acetonitrile.^22^ We expect this mechanism to be of more general validity, not just dictating the response of nitrile groups to an electrostatic dipole but also acting in a similar fashion in diazo and azide groups.

### D. HCN-dipole interaction in perpendicular arrangement

Lastly, we present simulated results on the HCN molecule in the presence of a dipole that is arranged perpendicularly to the CN bond. The loss spectrum is calculated by placing the dipole 2 Å away from the midpoint of the CN bond. This dipole arrangement can be envisioned for structurally fairly well-defined environments such as proteins, while in solutions, solvent molecules with an intrinsic dipole would orient antiparallel to the nitrile group. We note that in aqueous solution, partial HCN dissociation requires a decreased pH value to shift the equilibrium to HCN. Acetonitrile is an experimental alternative.^46,47^ but as for the purpose of establishing an understanding of electrostatic sensing on a fundamental level and relating structural degrees of freedom to valence charge distributions, the localized nature of excitations of the nitrile group allows transferring direct conclusions from HCN to similar nitrile systems.

**FIG. 6.**
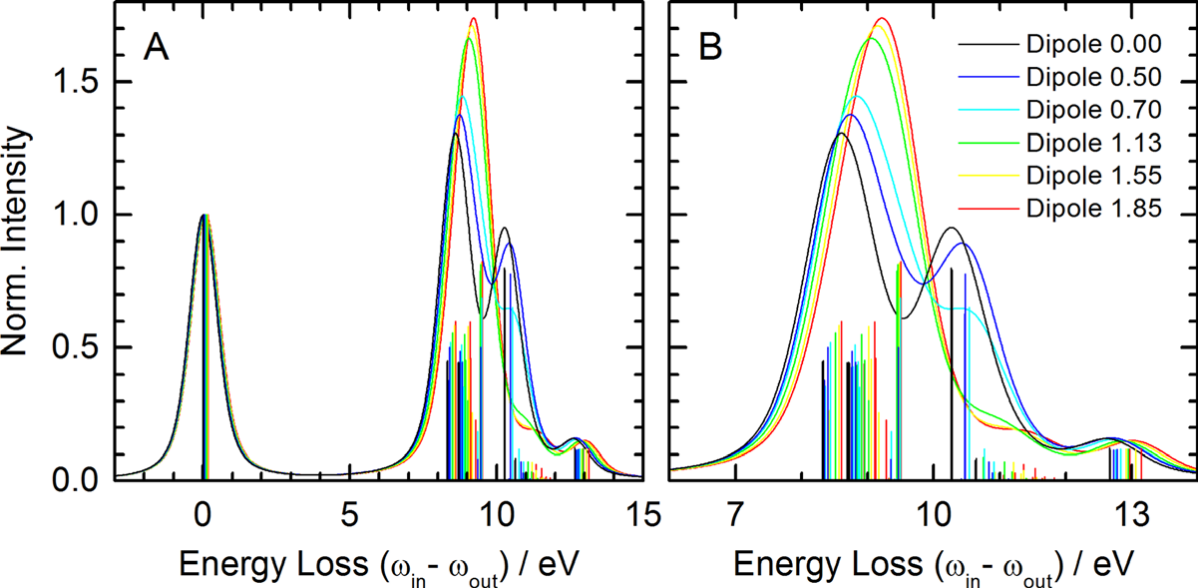
Simulated N-1 s loss spectra of HCN in the presence of an equatorial dipole of increasing strength as indicated in the legend. (a) Computed energy loss range. (b) Energy loss region with largest spectral changes. Vertical lines indicate transitions without broadening.

The dipole in a perpendicular arrangement is breaking the molecular symmetry, thereby re-hybridizing the nitrile's *π* and *σ* orbitals. The computed loss spectra are shown in Fig. 6. A very small negligible shift of the elastic line can be observed with respect to the dipole-free case. The behavior of the *σ* and *π* orbitals leads to an increasingly complex loss spectrum as a consequence of the symmetry loss. The transitions originating from *π* orbitals split as is evident from comparison of the transitions in Fig. 4(b) with those in Fig. 6(b) at 8–9 eV energy loss. The decrease in symmetry affects the *π*_1_ orbital facing the dipole in an manner opposite to that of the *π*_2_ and *σ* orbitals resulting in the following trends in the loss spectra: (i) the loss transitions associated with the two $\pi \to \pi^{*}$ transition at 8.3 and 8.7 eV shift to higher energy monotonically by about 0.1 eV/D. (ii) The $\sigma \to \pi^{*}$ transition at 10.3 eV migrates to higher energy and rapidly diminishes with increasing dipole strength while also changing its character to a $\pi_{1}\to$ LUMO + 2 transition. (iii) A $\pi_{1}\to$ LUMO + 2 transition of negligible oscillator strength grows in with increasing dipole strength at 9.3 eV, which conversely changes its character to a $\sigma \to \pi^{*}$ transition to become the dominant transition of the loss spectrum. We note that it is indeed a very abrupt change in transition character and not a gradual spectral migration.

**FIG. 7.**
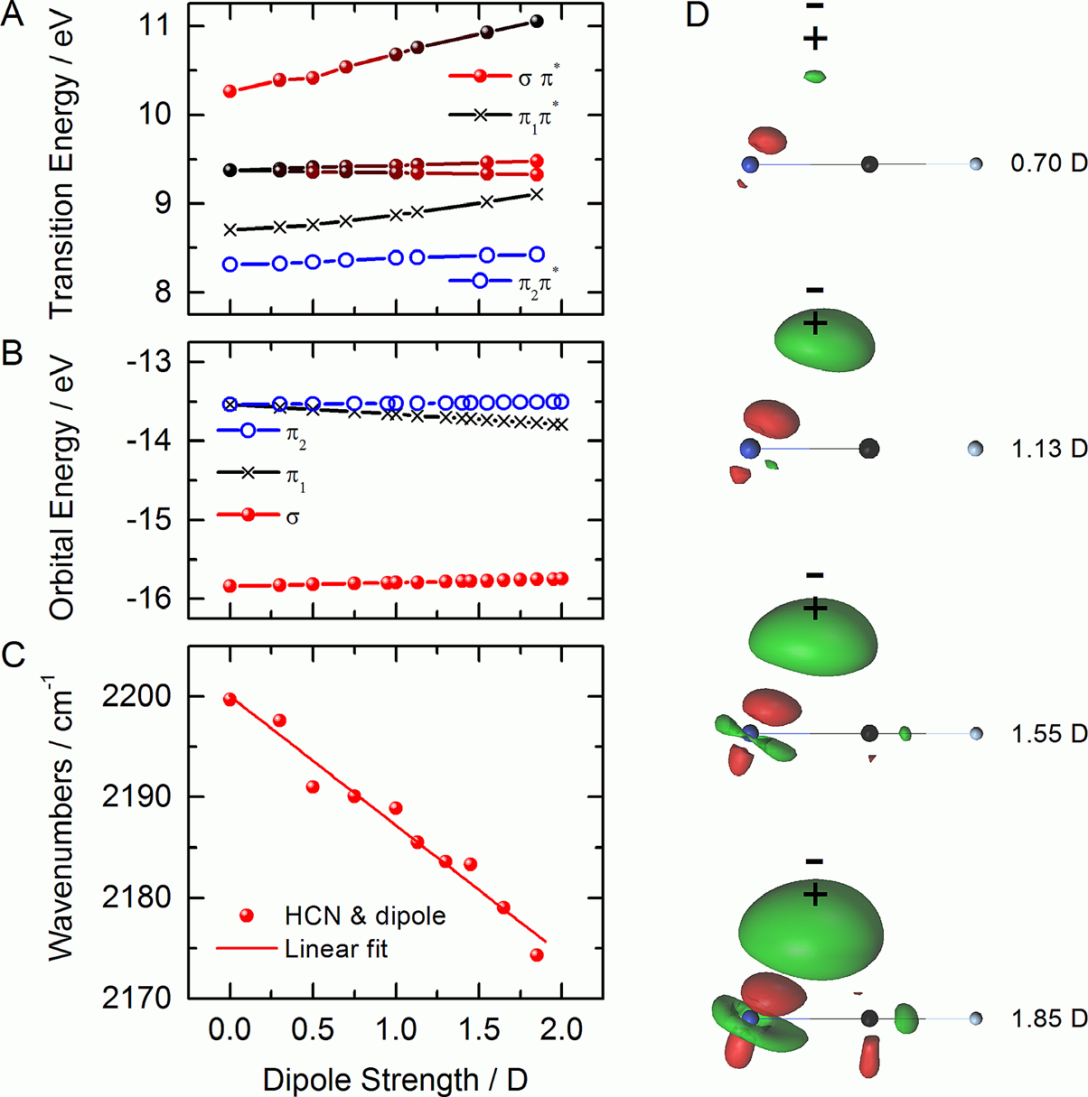
**(**a**)** Energies of valence excitation as a function of dipole strength. (b) Hartree-Fock orbital energies of the HOMO-*π* and HOMO-*σ* orbitals as a function of applied dipole strength. (c) Computed CN stretching frequency of HCN in harmonic approximation as a function of dipole strength. (d) Isodensity surfaces (at 0.005 value) of differential charge density between isolated HCN and HCN in the presence of a perpendicular dipole. Green regions denote positive (increased) and red regions denote negative (decreased) charge density.

In order to understand this behavior, valence excitation energies between the ground electronic state and the low-lying excited states with intensity in the loss spectra are displayed in Fig. 7(a) and Hartree-Fock orbital energies as a function of the dipole strength are presented in Fig. 7(b). The symmetry change lifts the degeneracy of the *π* orbitals and engenders an energetic approach of the *σ* and *π*_1_. Structurally, the CN bond softens considerably as Fig. 7(c) reveals. The bond softening can be traced to the dipole-induced delocalization in charge density. We visualize this change in charge density again as isosurfaces of the total charge density difference between no dipole and a dipole of varying strength in Fig. 7(d). Thus, a very characteristic spectral reshaping of the loss spectrum characterizes a perpendicular arrangement that can act as a proxy for this kind of electrostatic environment in more complex systems. Lastly, we note that the somewhat artificial case of a water molecule placed in perpendicular arrangement to the HCN molecule is predicted to induce very similar changes to the electronic structure of HCN.

## III. CONCLUSIONS

The interaction of a nitrile group with one water molecule and an electric dipole has been investigated by utilizing the *ab initio* RASSCF theory to calculate RIXS and spectra and vibrational frequencies. In comparison to isolated HCN, we find spectral shifts indicative of lone pair orbital stabilization on the nitrogen atom of the HCN-water complex. The spectral features due to hydrogen bonding are further investigated by replacing water with an electric dipole. The shift of the CN stretching mode to higher frequency is found for both, a dipole and a hydrogen bond, in agreement with reported results.^22,64,65^ We conclude from these results that the effects of hydrogen bonding are mostly of electrostatic nature. These effects are therefore universal and not linked to particular ligands around the nitrile group. A dipole charge distribution aligned with the nitrile group lowers the energy of the *σ* orbital and leads to a strengthening of the CN bond via polarization of the *σ*-symmetry orbitals. This can be distinctly seen in the corresponding vibrational frequency, but also very clearly as a marked shift in the Raman UV line at 10.5 eV. The effect is the opposite when the dipole is placed perpendicular to the nitrile group, leading to a weakening of the CN bond. The dominant *σ* and *π* transitions are reshuffled and the transitions corresponding to relaxations from the *π* orbitals split because the molecular symmetry is broken by the electrostatic environment.

Our analysis approach links UV excitations of the electronic system to the molecular structure, which is important for understanding the interplay of electronic and nuclear degrees of freedom. This work also indicates that RIXS spectroscopy of nitrile groups may act as a sensitive and complimentary tool of hydrogen bonding and electrostatic environments in solvated systems or small model peptides. The electronic structure information can facilitate the interpretation and deepen the understanding of local dynamics where such vibrational reporters are employed. Further investigations of different nitrogen-containing functional groups and more complex electrostatic environments will follow.
